# Supplementary material for: The transcription factor PREP1(PKNOX1) regulates nuclear stiffness, the expression of LINC complex proteins and mechanotransduction
Source: Commun Biol. 2022 May 12;5:456. doi: 10.1038/s42003-022-03406-9 (PMC9098460; doi:10.1038/s42003-022-03406-9)

Supplementary Figure 1

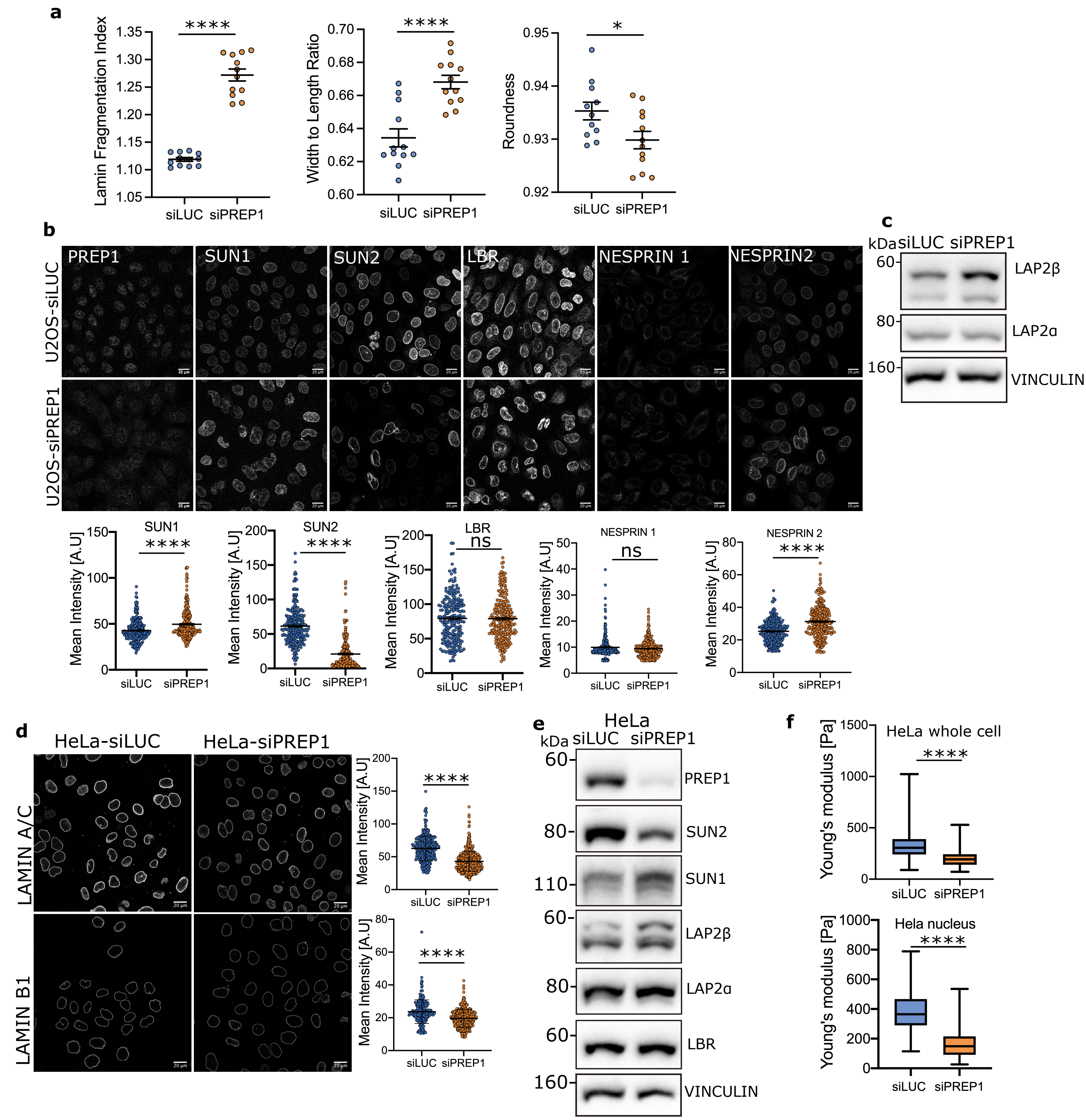

Supplementary Figure 1. PREP1 regulates nuclear envelope proteins. (a)Representative graphs which shows width to length ratio, roundness and lamin fragmentation in control siLUC vs PREP1 downregulated (siPREP1) cells. Each data point is an average of 50-200 nuclei in a single well. Total number of cells counted: siLUC=2036, siPREP1=1496. (b) Representative fluorescence images showing the expression of PREP1, SUN1, SUN2, LBR, NESPRIN1 and NESPRIN2 in siLUC and siPREP1 cells. Scale bar is 20 µm. Scatter plots below show the quantification of fluorescence intensity in siLUC and siPREP1 cells. N=245 (siLUC), 266 (siPREP1) for SUN2, N=252 (siLUC), 243 (siPREP1) for SUN1, N=216 (siLUC), 232 (siPREP1) for LBR, N=281 (siLUC), 352 (siPREP1) for NESPRIN 1, N=296 (siLUC), 298 (siPREP1) for NESPRIN2.Data is representative of three independent experiments. (c) A represetative westernblot showing LAP2β and LAP2α levels in siLUC and siPREP1 U2OS cells. (d) Representative images showing LAMIN B1 and LAMIN A/C levels in control (siLUC) and PREP1 depleted (siPREP1) HeLa cells. Scatter plots show the quantification of the fluorescence intensity. N=325 (siLUC), 636 (siPREP1) for LAMIN A/C, N=224 (siLUC), 440 (siPREP1) for LAMIN B1 analysis. Data is representative of three or more independent experiments. (e) Representative westernblot showing levels of various nuclear envelope proteins in siLUC and siPREP1 HeLa cells. (f) Elastic modulus (Youngs' modulus) assessed by AFM in control (siLUC) and PREP1 depleted (siPREP1) HeLa cells (upper panel) or isolated nuclei (lower panel). n=40 cells. Error bars in the graphs denote mean±SE

Supplementary Figure 2

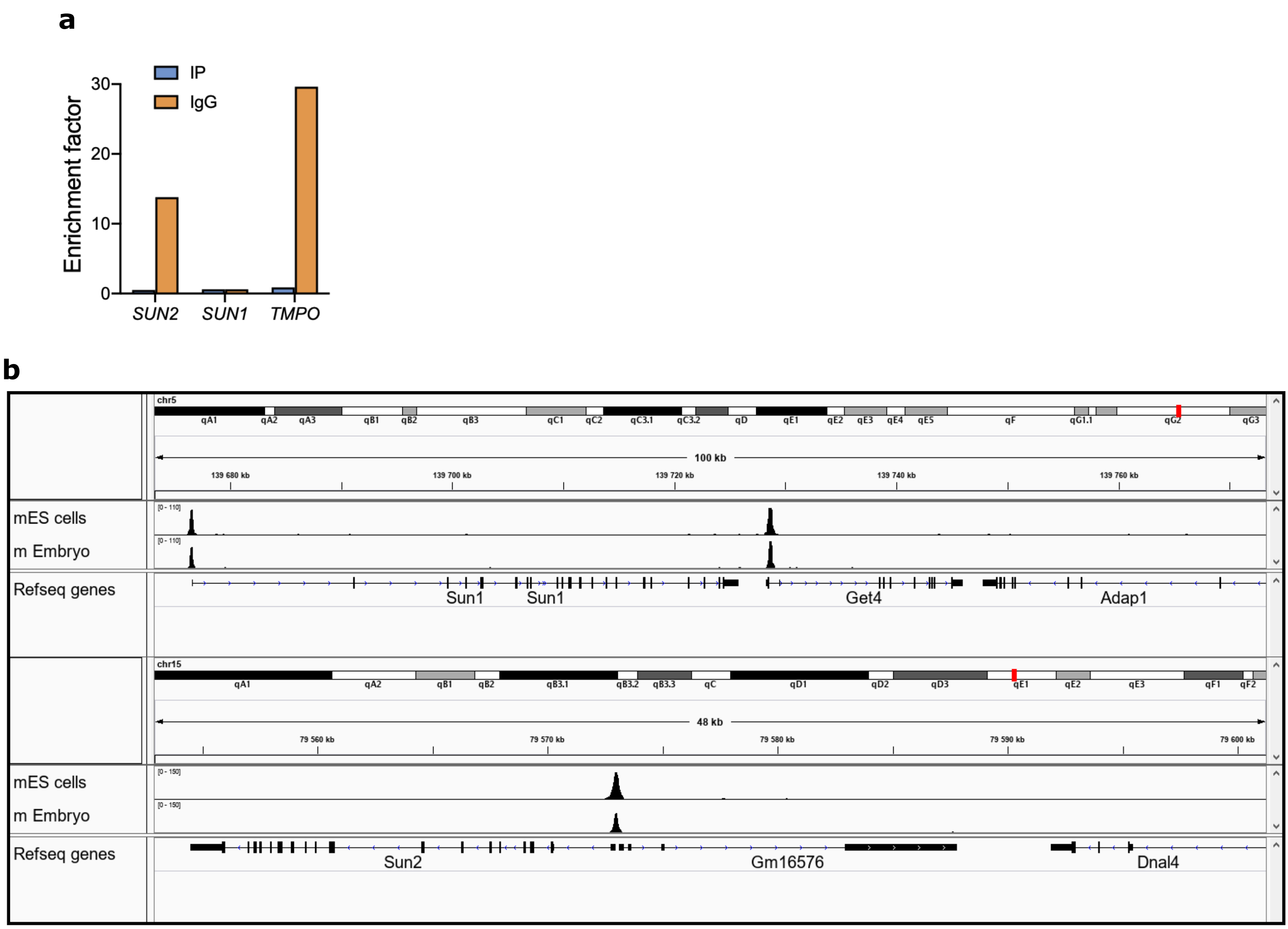

Supplementary Figure 2 (**a**). Chip-PCR data showing PREP1 enrichment at the regulatory regions of *SUN2* (promoter) (enhancer) and *TMPO*/*LAP2* (enhancer) in Hela cells. (**b**) PREP1 binding profiles (Big Wig files) to the loci of *Sun1* and *Sun2* genes in the IGV genome browser are shown. Cells used in the Chip-seq analysis are mouse embryonic stem cells (ES) and total cells from E11.5 mouse embryo trunk (embryo).

Supplementary Figure 3

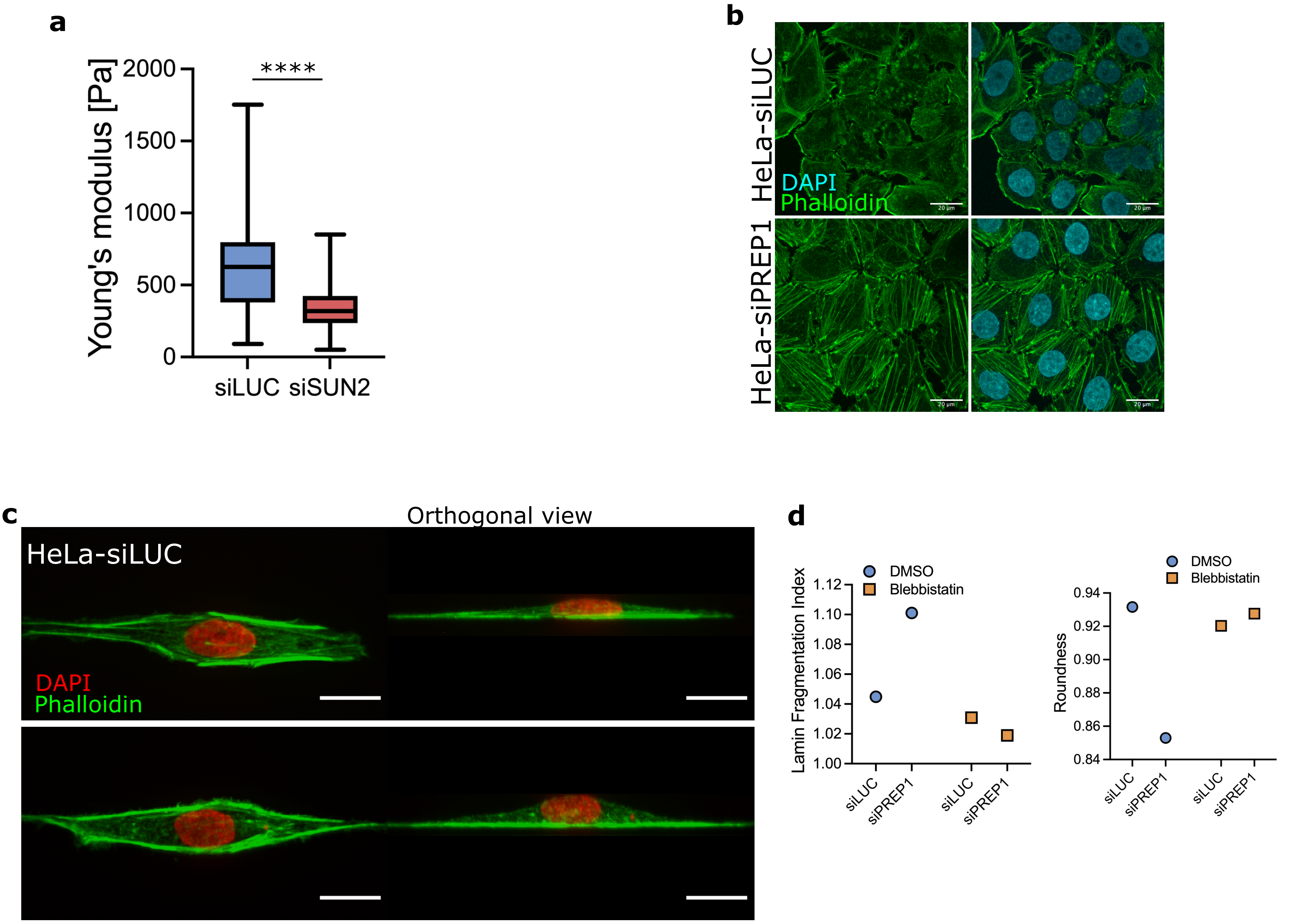

Supplementary Figure 3 (**a**) Box plot showing Youngs' modulus measured by AFM in control (siLUC) and SUN2 depleted (siSUN2) U2OS cells. N=40 cells. $p<0.0001$. (**b**) Representative confocal images showing F-actin (phalloidin) and nucleus (DAPI) in control (siLUC) and PREP1 depleted (siPREP1) HeLa cells. Scale bar is 20μm. (**c**) Maximum projection of confocal stacks of F-actin (phalloidin)in green and nucleus (DAPI) in red in siLUC and siPREP1 HeLa cells grown in fibronectin coated micropatterns. Left panel shows orthogonal view of the same cells. Scale bar is 40μm. (**d**) Scatter plots show mean lamin fragmentation index and mean roundness of nuclei in siLUC or siPREP1 U2OS cells. N= 57 (siLUC DMSO), 70 (siLUC Blebbistatin), 66 (siPREP1 DMSO) and 106 (siPREP1 Blebbistatin) cells.

Supplementary Figure 4

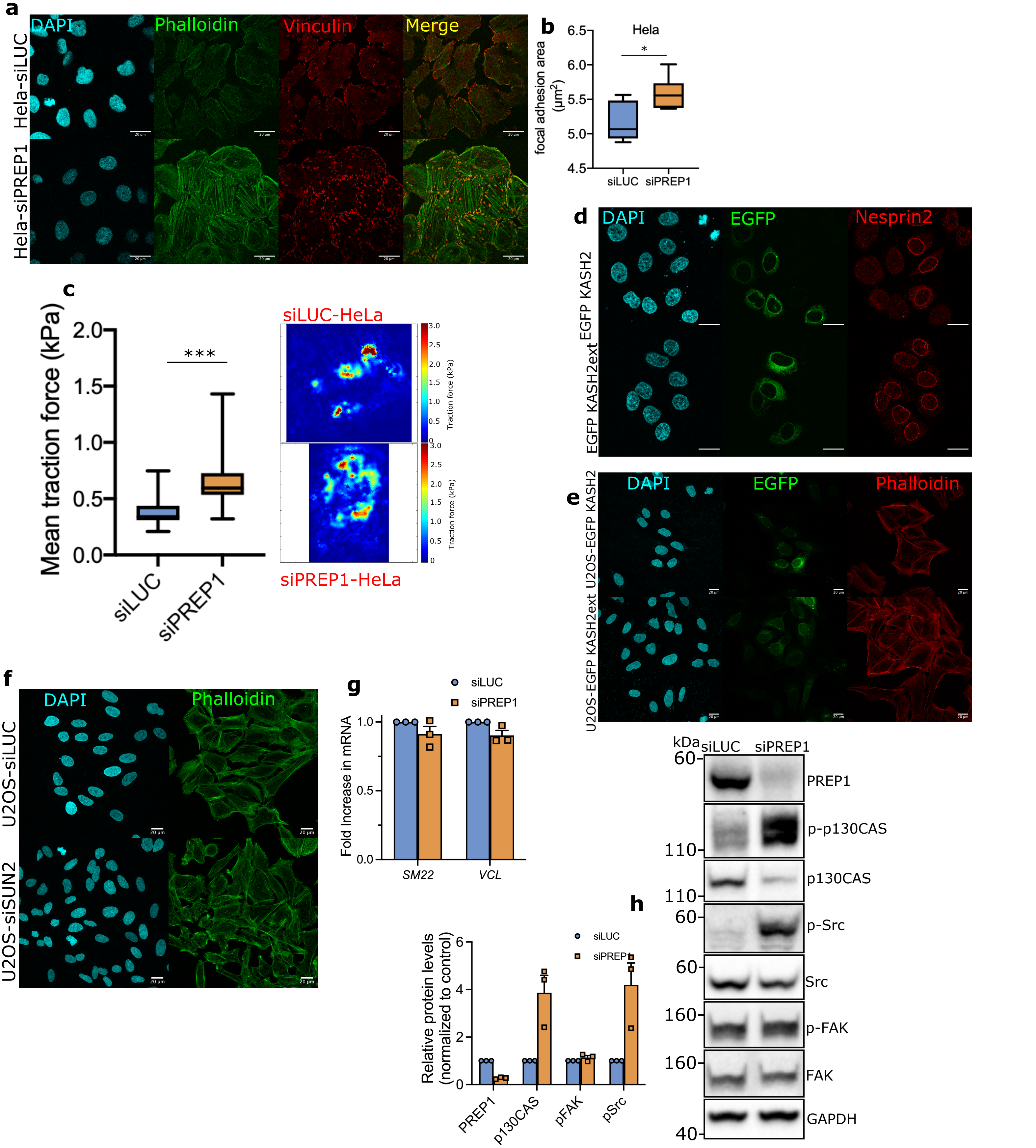

Supplementary Figure 4. (a)Representative images showing nucleus (DAPI) F-actin (phalloidin) and vinculin in siLUC and siPREP1 HeLa cells. (b) The box pot shows quantification of vinculin foci area in cells as in (a). Data derived from two independent experiments. N=252 (siLUC) and 331 (siPREP1) cells. (c)Traction force heat-map images of control (siLUC) and PREP1 depleted (siPREP1) HeLa cells. The box plot shows mean traction force in kPa by siLUC or siPREP1 cells. The data is derived from three independent experiments. N= 15 (siLUC) and 29 (siPREP1) ells. p=0.0002. (d) Confocal image showing Nesprin 2 localization in HeLa cells transfected with EGFP-KASH2 and EGFP-KASH2ext constructs. (e) F-actin (phalloidin) staining in U2OS cells transfected with constructs as in (d). (f) Confocal image showing F-actin (phalloidin) in control and SUN2 depleted U2OS cells.(g) Bar graph showing mRNA levels of SM22 and Vinculin (VCL) in control (siLUC) and PREP1 depleted (siPREP1) U2OS cells. (h) A representative westernblot showing phosphorylation status of p130CAS, Src and FAK in siLUC and siPREP1 HeLa cells. Bar graph shows relative protein levels in siPREP1cells with respect to siLUC. Error bars in graphs represent mean±SE. Scale bars show 20μ in the images

Supplementary Figure 5

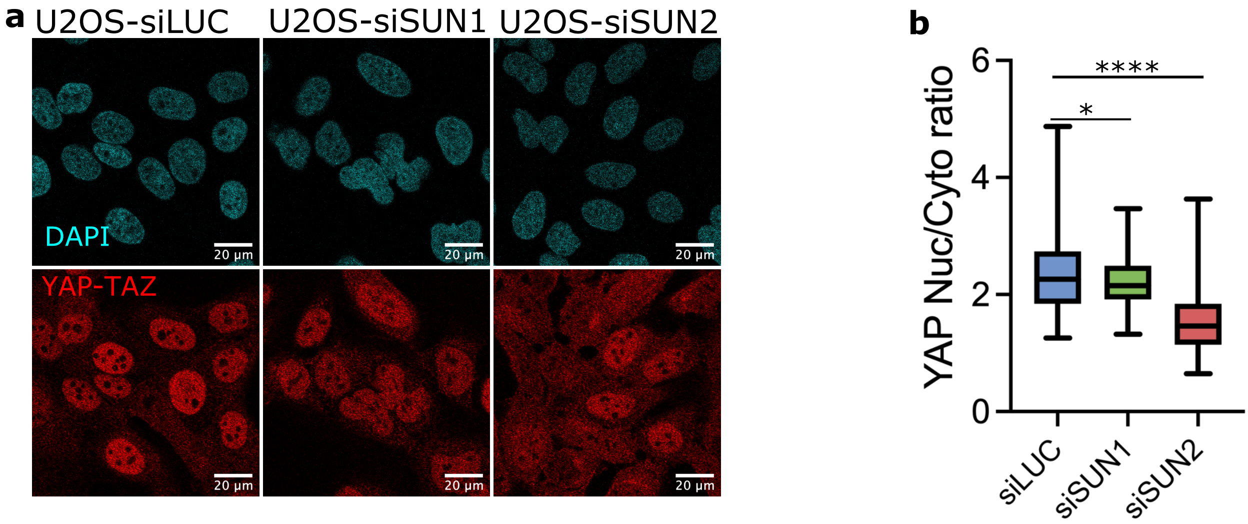

Supplementary Figure 5. (**a**) Representative confocal images showing nucleus (DAPI-cyan) and YAP-TAZ (Red) in control (siLUC), SUN1 depleted (siSUN1) and SUN2 depleted (siSUN2) U2OS cells. Scale bar is 20µm. (B) Bar graph showing nuclear to cytoplasmic ratio of YAP-TAZ detected by the total YAP antibody. N=82 cells for siLUC and 100 for both siSUN1 and siSUN2. p=0.046 (*), p<0.0001 (****)

Supplementary Figure 6

Fig 1e

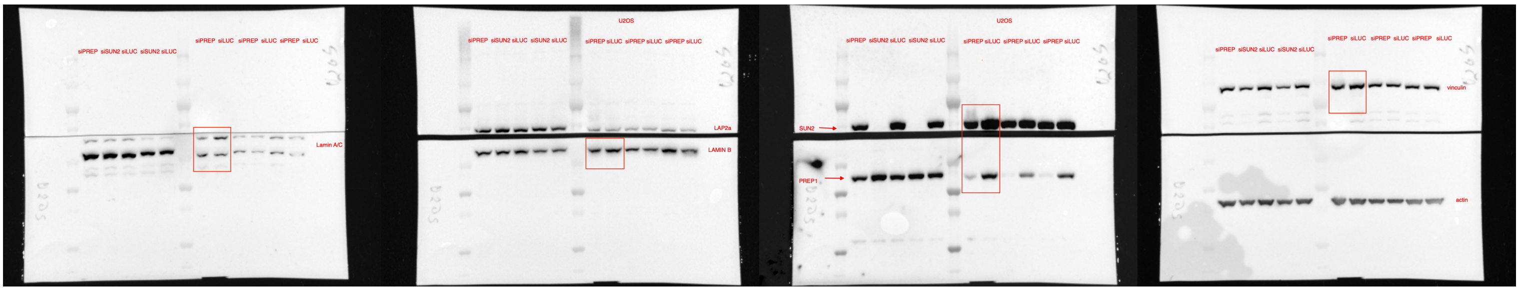

Figure 1F and Supplementary Fig 1c

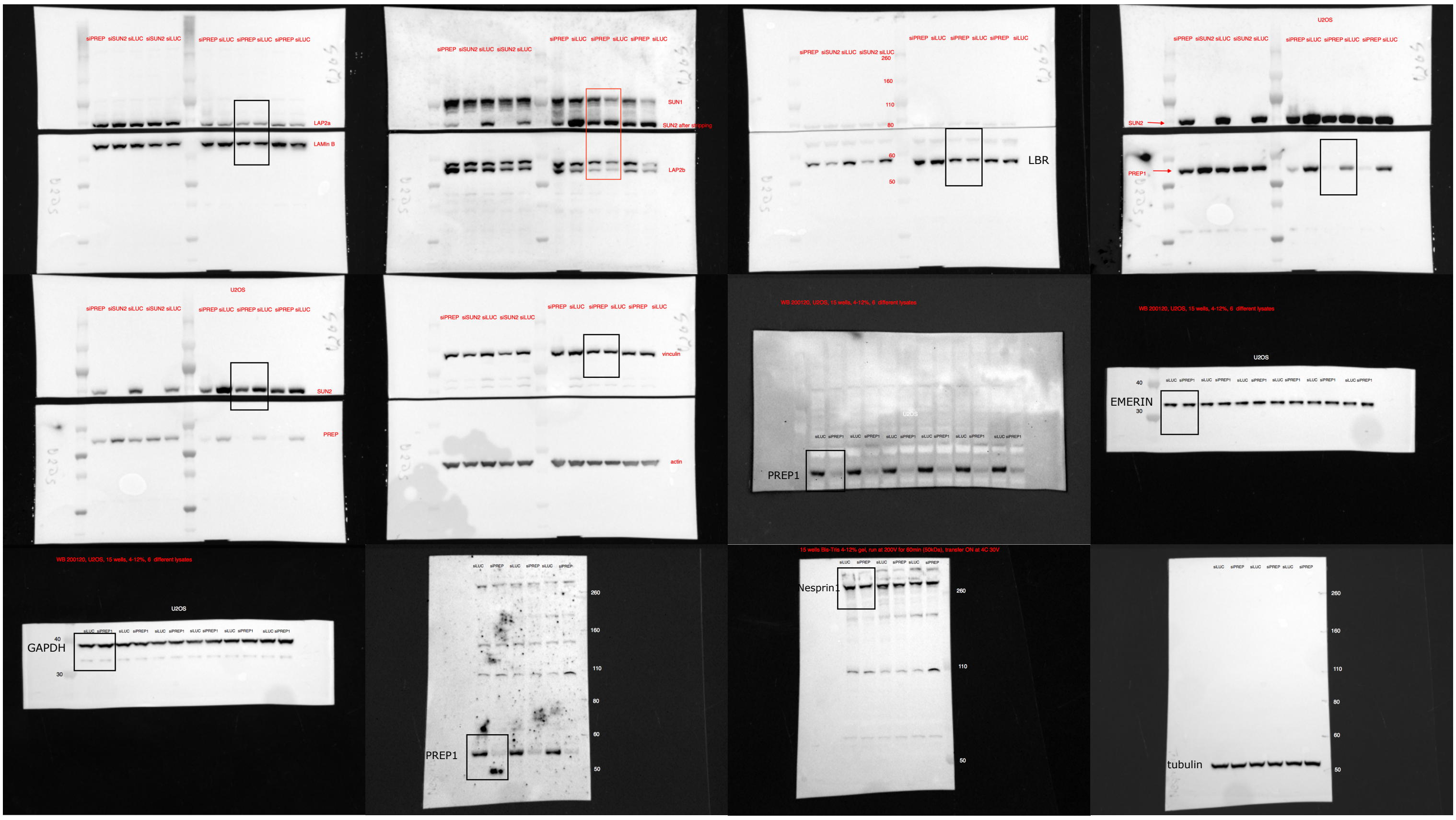

Fig 1g

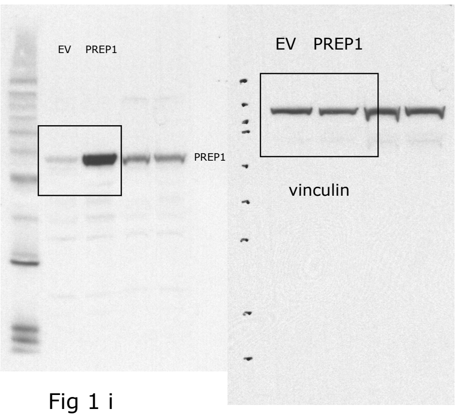

Fig 1 i

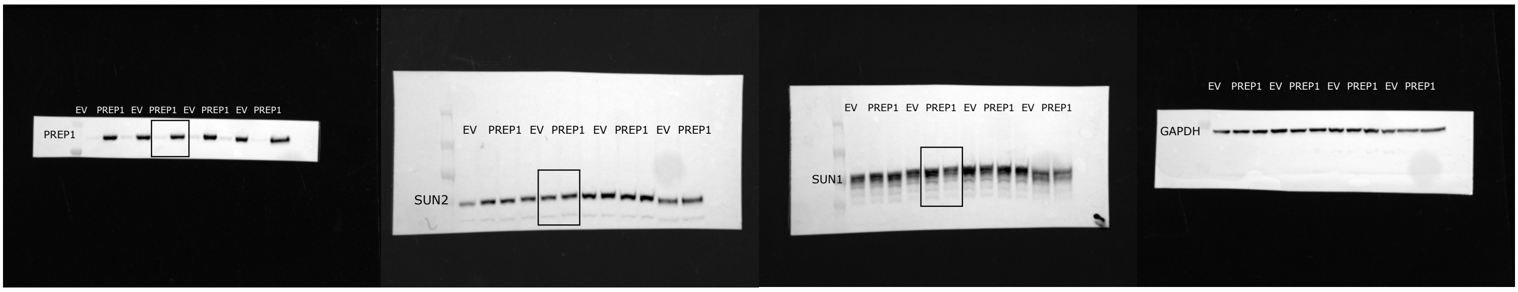

Supplementary Figure 6

Fig. 3a 3b

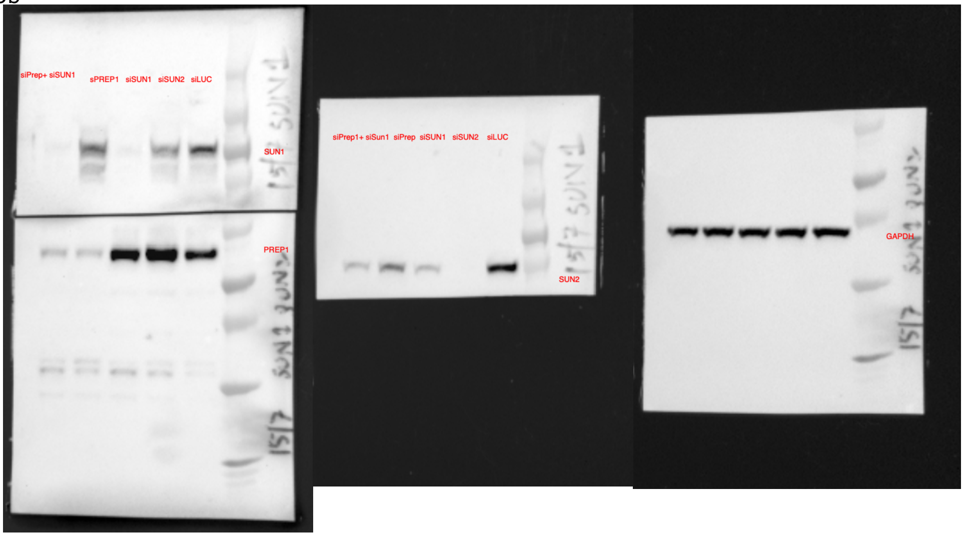

Fig. 4f and Supplementary figure 4h

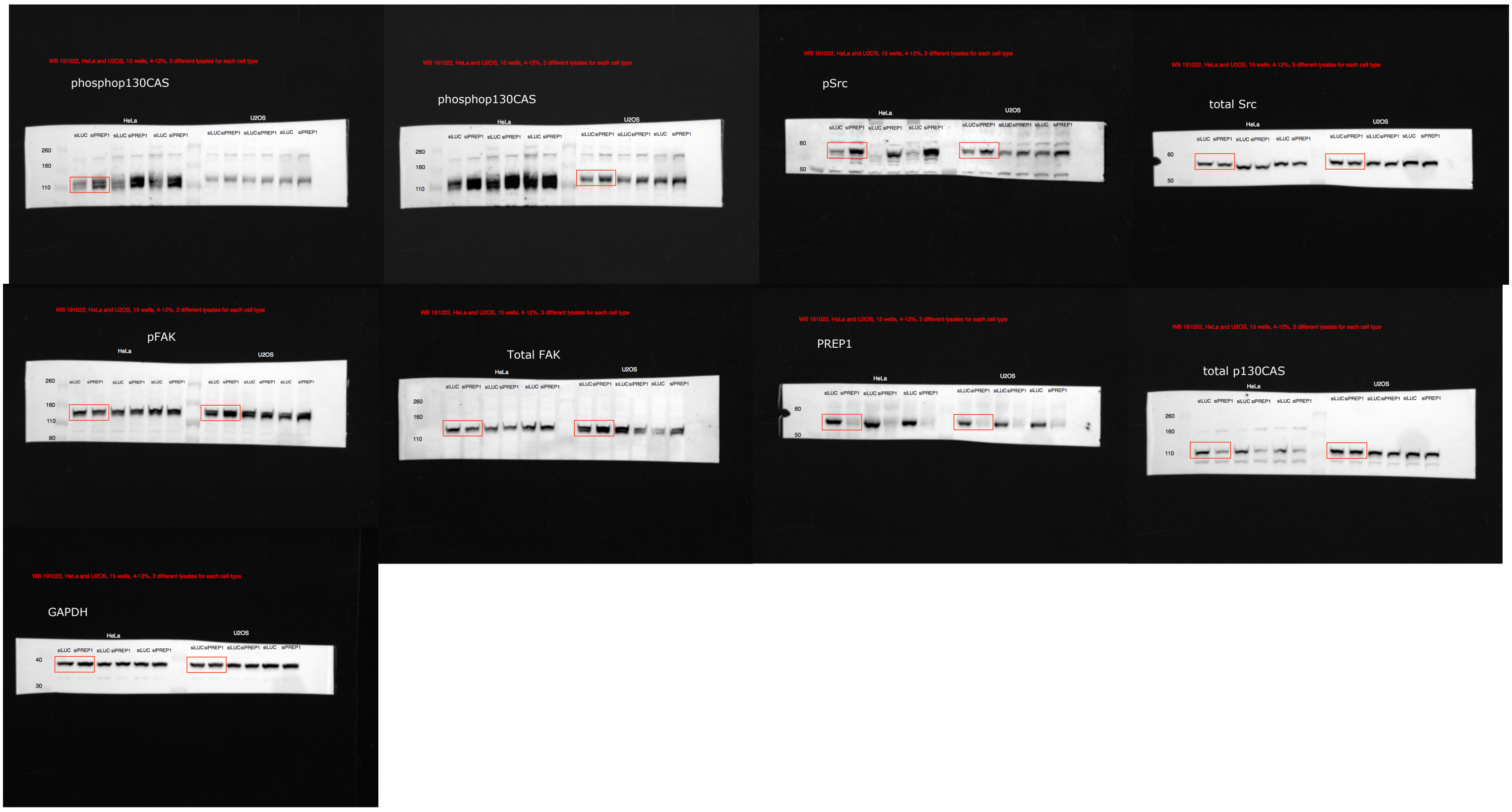

Supplementary figure 1e

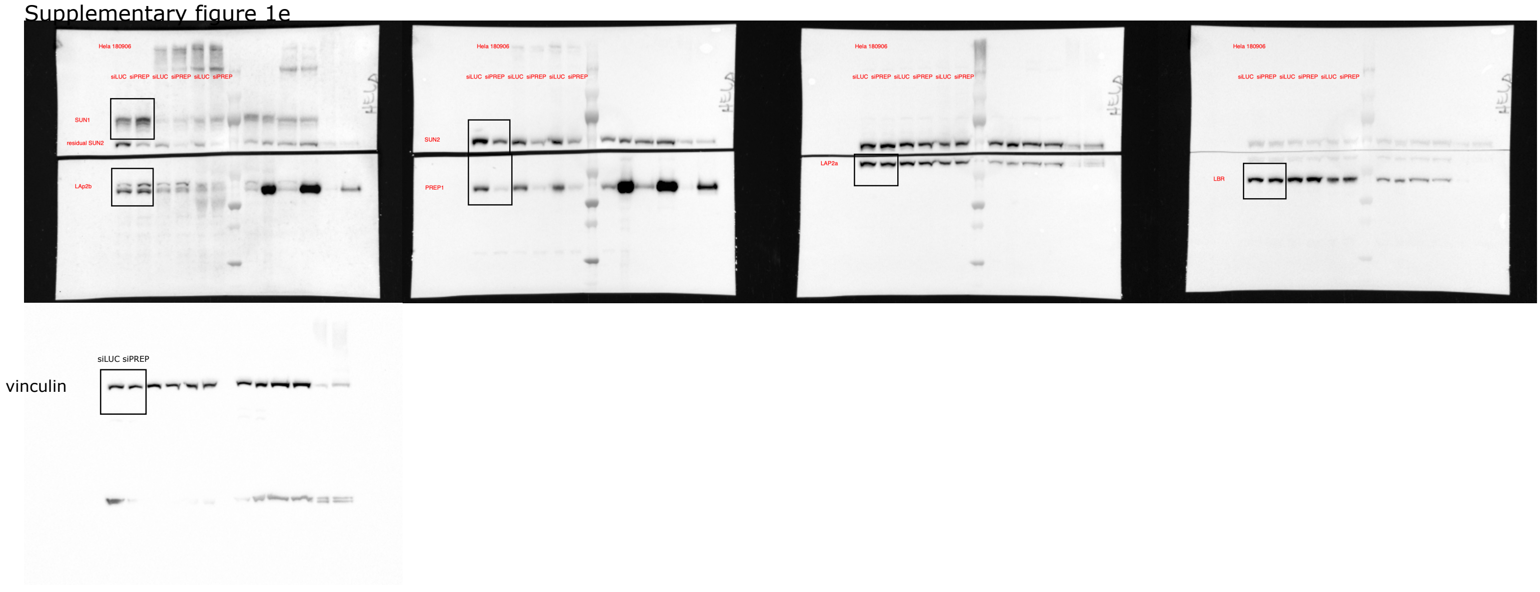

Supplementary Figure 6

Fig. 5c

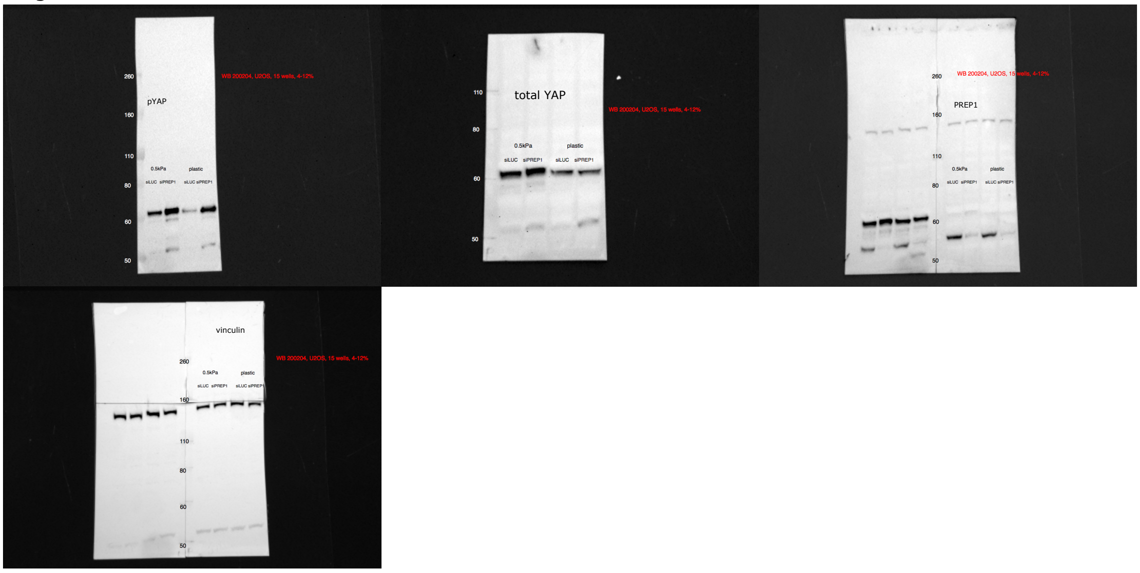

Fig. 5d

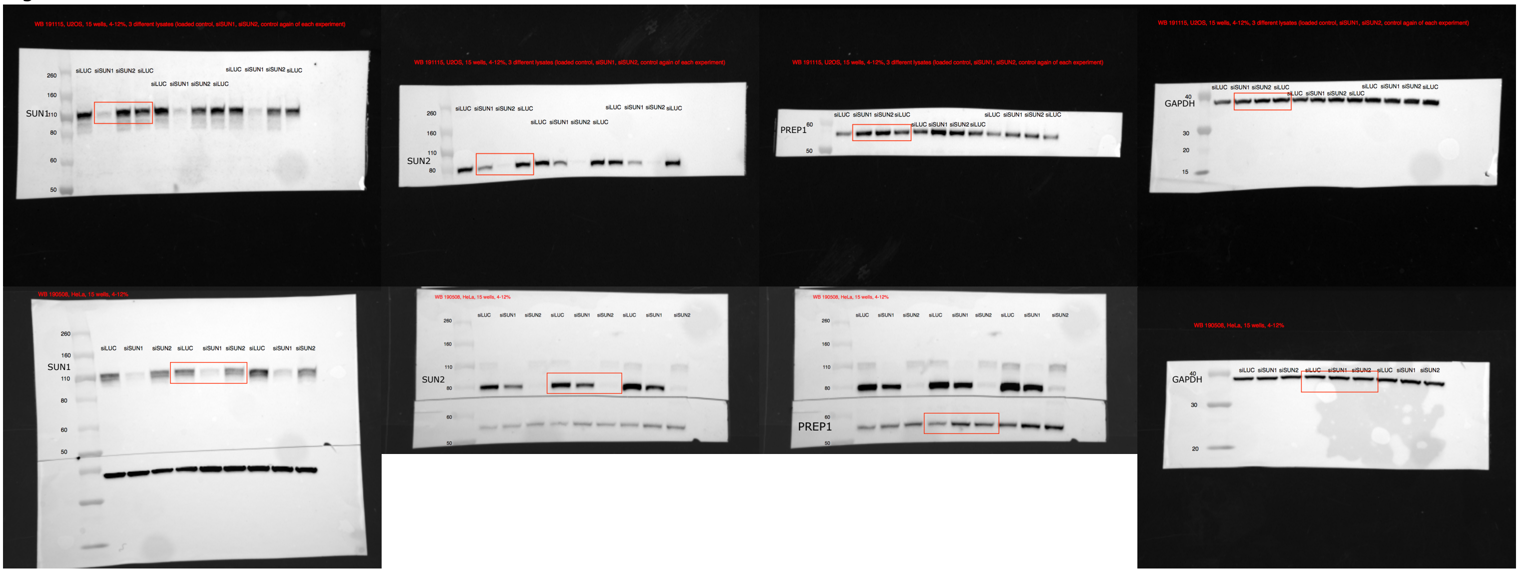

Fig. 5f

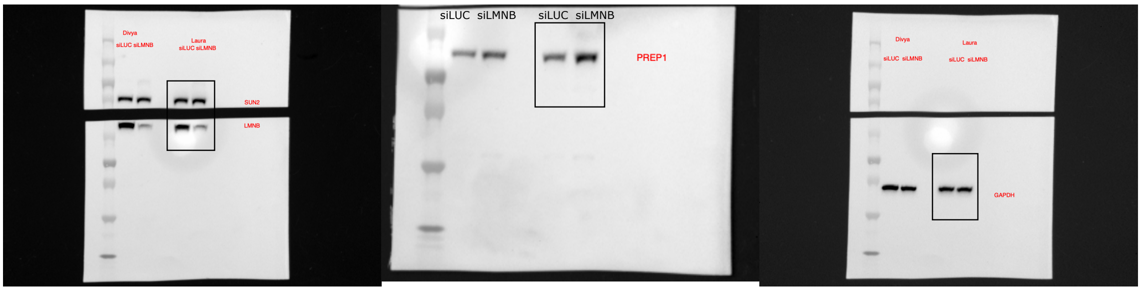

Supplement: Supplementary file 2 — Supplementary Information [file 42003_2022_3406_MOESM2_ESM.pdf]
