# Supplementary material for: The transcription factor PREP1(PKNOX1) regulates nuclear stiffness, the expression of LINC complex proteins and mechanotransduction
Source: Commun Biol. 2022 May 12;5:456. doi: 10.1038/s42003-022-03406-9 (PMC9098460; doi:10.1038/s42003-022-03406-9)
Supplement: Supplementary file 3 — Description of Additional Supplementary Files [file 42003_2022_3406_MOESM3_ESM.pdf]

Description of Additional Supplementary Files

File name: Supplementary Data 1
Description: The source data for all the graphs prepared for the manuscript.
