# Supplementary material for: The transcription factor PREP1(PKNOX1) regulates nuclear stiffness, the expression of LINC complex proteins and mechanotransduction
Source: Commun Biol. 2022 May 12;5:456. doi: 10.1038/s42003-022-03406-9 (PMC9098460; doi:10.1038/s42003-022-03406-9)
Supplement: Supplementary file 5 — Reporting Summary [file 42003_2022_3406_MOESM5_ESM.pdf]

nature research

Corresponding author(s): Francesco Blasi, Divya Purushothaman

Last updated by author(s): March 13, 2022

Reporting Summary

Nature Research wishes to improve the reproducibility of the work that we publish. This form provides structure for consistency and transparency in reporting. For further information on Nature Research policies, see our Editorial Policies and the Editorial Policy Checklist.

Statistics

For all statistical analyses, confirm that the following items are present in the figure legend, table legend, main text, or Methods section.

n/a	Confirmed	
☐	☒	The exact sample size (n) for each experimental group/condition, given as a discrete number and unit of measurement
☐	☒	A statement on whether measurements were taken from distinct samples or whether the same sample was measured repeatedly
☐	☒	The statistical test(s) used AND whether they are one- or two-sided<br>*Only common tests should be described solely by name; describe more complex techniques in the Methods section.*
☒	☐	A description of all covariates tested
☒	☐	A description of any assumptions or corrections, such as tests of normality and adjustment for multiple comparisons
☐	☒	A full description of the statistical parameters including central tendency (e.g. means) or other basic estimates (e.g. regression coefficient) AND variation (e.g. standard deviation) or associated estimates of uncertainty (e.g. confidence intervals)
☐	☒	For null hypothesis testing, the test statistic (e.g. F, t, r) with confidence intervals, effect sizes, degrees of freedom and P value noted<br>*Give P values as exact values whenever suitable.*
☒	☐	For Bayesian analysis, information on the choice of priors and Markov chain Monte Carlo settings
☒	☐	For hierarchical and complex designs, identification of the appropriate level for tests and full reporting of outcomes
☒	☐	Estimates of effect sizes (e.g. Cohen's d, Pearson's r), indicating how they were calculated

Our web collection on statistics for biologists contains articles on many of the points above.

Software and code

Policy information about availability of computer code

Data collection	Leica LCS (version 2.6.1), Leica LAS AF (version 2.7.3.9723), Perkin Elmer Volocity (version 6.3)
Data analysis	R studio (http://www.r-project.org/) , custom-built ImageJ (version 1.52i) plugins, GraphPad Prism (version 7.0 and 8.0), Perkin Elmer Acapella.

For manuscripts utilizing custom algorithms or software that are central to the research but not yet described in published literature, software must be made available to editors and reviewers. We strongly encourage code deposition in a community repository (e.g. GitHub). See the Nature Research guidelines for submitting code & software for further information.

Data

Policy information about availability of data

All manuscripts must include a data availability statement. This statement should provide the following information, where applicable:

- Accession codes, unique identifiers, or web links for publicly available datasets
- A list of figures that have associated raw data
- A description of any restrictions on data availability

Chip-seq data on human cardiac mesenchymal stem cells generated during this study have been deposited at GEO and is retrievable with the accession code GSE160286. Chip-seq data on Hela cells (accession code GSE101776) , mouse embryos (accession code GSE39609) and mouse embryonic stem cells (accession code GSE63282) are available at GEO database . All other data supporting the findings of this study are available within the article and its Supplementary Information files or from the corresponding authors upon reasonable request

Field-specific reporting

Please select the one below that is the best fit for your research. If you are not sure, read the appropriate sections before making your selection.

☒ Life sciences ☐ Behavioural & social sciences ☐ Ecological, evolutionary & environmental sciences

For a reference copy of the document with all sections, see nature.com/documents/nr-reporting-summary-flat.pdf

Life sciences study design

All studies must disclose on these points even when the disclosure is negative.

Sample size	Sufficient replicates were performed for statistical significance. Sample size for each experiment is indicated.
Data exclusions	No data exclusions were done
Replication	All the data were successfully reproduced in independent experiments and is reproducible
Randomization	N/A
Blinding	N/A

Reporting for specific materials, systems and methods

We require information from authors about some types of materials, experimental systems and methods used in many studies. Here, indicate whether each material, system or method listed is relevant to your study. If you are not sure if a list item applies to your research, read the appropriate section before selecting a response.

Materials & experimental systems

n/a	Involved in the study	
☐	☒	Antibodies
☐	☒	Eukaryotic cell lines
☒	☐	Palaeontology and archaeology
☒	☐	Animals and other organisms
☒	☐	Human research participants
☒	☐	Clinical data
☒	☐	Dual use research of concern

Methods

n/a	Involved in the study	
☐	☒	ChIP-seq
☒	☐	Flow cytometry
☒	☐	MRI-based neuroimaging

Antibodies

Antibodies used

Emerin (4G5) Monoclonal mouse NCL-EMERIN Leica Biosystems
FAK (B-8) Monoclonal mouse sc-271195 Santa Cruz Biotechnology
FAK-phospho Tyr397 (2D11) Monoclonal mouse sc-81493 Santa Cruz Biotechnology
GAPDH (6C5) Monoclonal mouse sc-32233 Santa Cruz Biotechnology
Lamin A/C (636) Monoclonal mouse sc7292 Santa Cruz Biotechnology
Lamin B1 Polyclonal rabbit ab16048 Abcam
Lamin B1 (C-20) polyclonal goat sc6216 Santa Cruz Biotechnology
Lamin B Receptor (clone E398L) monoclonal rabbit ab32535 Abcam
LAP2 alpha rabbit Polyclonal ab5162 Abcam
LAP2 beta (clone LAP106) monolonal mouse L6043 Sigma
Nesprin 1 MANNES1A(7A12) Monoclonal mouse MA5-18077 Thermo Scientific
Nesprin 2 Polyclonal rabbit IQ565 Immuquest
Nesprin 2 K20-478-5 Monoclonal mouse MA5-18075 Thermo Scientific
p130 Cas (E1L9H) Monoclonal rabbit 13846 Cell Signaling Technology
p130 Cas-phospho Tyr410 Polyclonal rabbit 4011 Cell Signaling Technology
PREP1 (clone B2) Monoclonal mouse sc25282 Santa Cruz Biotechnology
Src (GD-11) Monoclonal mouse 05-184 Merck Millipore
Src-phospho Tyr416 (D49G4) Monoclonal rabbit 6943 Cell Signaling Technology.
SUN1 EPR6554 Monoclonal rabbit ab124770 Abcam
SUN2 EPR6557 Monoclonal rabbit ab124916 Abcam
Tubulin (TUB 2.1) Monoclonal mouse T4026 Sigma
Vinculin (hVIN-1) Monoclonal mouse V9131 Sigma
YAP (63.7) Monoclonal mouse sc101199 Santa Cruz Biotechnology
YAP-phospho Ser127 Polyclonal rabbit 4911 Cell Signaling Technology

Validation	Validation as provided by the manufacturer's on their website (above)<br>Additionally PREP1, SUN1, SUN2 and LAMIN B1 (Abcam) antibodies were validated in the manuscript using western blot.

Eukaryotic cell lines

Policy information about cell lines

Cell line source(s)	HeLa , A549 and U2OS cell lines were obtained from ATCC. Human primary cardiac mesenchymal stromal cells were obtained from the right ventricular free wall of cadaveric donors within 24 hours of death (accidental death, healthy subjects) and propagated in TMES medium as reported previously in Pilato, C. A. et al. Isolation and characterization of cardiac mesenchymal stromal cells from endomyocardial bioptic samples of arrhythmogenic cardiomyopathy patients. J. Vis. Exp. 2018, (2018).
Authentication	Cell line authentication of HeLa, A549 and U2OS cell lines were performed by genetic profiling using polymorphic short tandem repeat (STR) loci.
Mycoplasma contamination	Mycoplasma testing was regularly conducted to assure that all cells used were mycoplasma free at all times.
Commonly misidentified lines<br>(See ICLAC register)	No misidentified cell lines were used in this study.

ChIP-seq

Data deposition

☒ Confirm that both raw and final processed data have been deposited in a public database such as GEO.

☒ Confirm that you have deposited or provided access to graph files (e.g. BED files) for the called peaks.

Data access links<br>*May remain private before publication.*	https://www.ncbi.nlm.nih.gov/geo/query/acc.cgi?&acc=GSE160286<br>secure token:odoxcuiippqrdgr
Files in database submission	178R_001_IonXpress_rawlib.fastq, 178R_002_IonXpress_rawlib.fastq, 182R_011_IonXpress_rawlib.fastq, 182R_012_IonXpress_rawlib.fastq, 178R_001_IonXpress_rawlib.fq.srt.bw, 178R_002_IonXpress_rawlib.fq.srt.bw, 182R_011.bw, 182R_012.bw
Genome browser session<br>(e.g. UCSC)	GSE160286

Methodology

Replicates	Sample 1: CMSC donor1-pknox1 Chip, Sample 2: CMSC donor1 input DNA, Sample 3: CMSC donor2-pknox1 Chip, Sample 4: CMSC donor2 input DNA
Sequencing depth	Sample 1: 44 million reads.<br>Sample 2: 33,5 million reads.<br>Sample 3: 47.7 million reads<br>Sample 4: 47.8 million reads
Antibodies	PREP1 (N15) Polyclonal rabbit sc6245 Santa Cruz Biotechnology
Peak calling parameters	MACS version 2.0.10 with default parameters
Data quality	FDR<0.05; p-value <0,00001
Software	Reads were aligned to the hg19 genome using Bowtie v.0.12.8, allowing up to two mismatches per read and discarding reads with more than one mapping.<br>Duplicate reads were removed using Samtools rmdup v 0.1.18.<br>To identify enriched domains we used MACS version 2.0.10 with default parameters.
